# Increasing Upstream Chromatin Long–Range Interactions May Favor Induction of Circular RNAs in LysoPC-Activated Human Aortic Endothelial Cells

**DOI:** 10.3389/fphys.2019.00433

**Published:** 2019-04-18

**Authors:** Angus Li, Yu Sun, Charles Drummer, Yifan Lu, Daohai Yu, Yan Zhou, Xinyuan Li, Simone J. Pearson, Candice Johnson, Catherine Yu, William Y. Yang, Kevin Mastascusa, Xiaohua Jiang, Jianxin Sun, Thomas Rogers, Wenhui Hu, Hong Wang, Xiaofeng Yang

**Affiliations:** ^1^Center for Metabolic Disease Research, Lewis Katz School of Medicine, Temple University, Philadelphia, PA, United States; ^2^Department of Biomedical Engineering, Pratt School of Engineering, Duke University, Durham, NC, United States; ^3^Department of Clinical Sciences, Lewis Katz School of Medicine, Temple University, Philadelphia, PA, United States; ^4^Biostatistics and Bioinformatics Facility, Fox Chase Cancer Center, Temple Health, Philadelphia, PA, United States; ^5^Geisinger Commonwealth School of Medicine, Scranton, PA, United States; ^6^Center for Translational Medicine, Department of Medicine, Sidney Kimmel Medical College, Philadelphia University – Thomas Jefferson University, Philadelphia, PA, United States; ^7^Center for Inflammation, Translational and Clinical Lung Research, Lewis Katz School of Medicine, Temple University, Philadelphia, PA, United States

**Keywords:** circular RNAs, human aortic endothelial cell activation, proatherogenic lipid lysophosphatidylcholine, RNA-Seq, chromatin long–range interaction

## Abstract

Circular RNAs (circRNAs) are non-coding RNAs that form covalently closed continuous loops, and act as gene regulators in physiological and disease conditions. To test our hypothesis that proatherogenic lipid lysophosphatidylcholine (LPC) induce a set of circRNAs in human aortic endothelial cell (HAEC) activation, we performed circRNA analysis by searching our RNA-Seq data from LPC-activated HAECs, and found: (1) LPC induces significant modulation of 77 newly characterized cirRNAs, among which 47 circRNAs (61%) are upregulated; (2) 34 (72%) out of 47 upregulated circRNAs are upregulated when the corresponding mRNAs are downregulated, suggesting that the majority of circRNAs are upregulated presumably via LPC-induced “abnormal splicing” when the canonical splicing for generation of corresponding mRNAs is suppressed; (3) Upregulation of 47 circRNAs is temporally associated with mRNAs-mediated LPC-upregulated cholesterol synthesis-SREBP2 pathway and LPC-downregulated TGF-β pathway; (4) Increase in upstream chromatin long-range interaction sites to circRNA related genes is associated with preferred circRNA generation over canonical splicing for mRNAs, suggesting that shifting chromatin long-range interaction sites from downstream to upstream may promote induction of a list of circRNAs in lysoPC-activated HAECs; (5) Six significantly changed circRNAs may have sponge functions for miRNAs; and (6) 74% significantly changed circRNAs contain open reading frames, suggesting that putative short proteins may interfere with the protein interaction-based signaling. Our findings have demonstrated for the first time that a new set of LPC-induced circRNAs may contribute to homeostasis in LPC-induced HAEC activation. These novel insights may lead to identifications of new therapeutic targets for treating metabolic cardiovascular diseases, inflammations, and cancers.

## Introduction

Cardiovascular diseases remain some of the most prevalent health challenges today. Predominant among these, atherosclerosis and resultant ischemic heart disease, stroke, and peripheral artery disease are estimated to cause 8.9 million deaths per year, making it the single most significant cause of death worldwide (Wang H. et al., 2016). We and others have reported that hyperlipidemia, together with other CVD risk factors, such as hyperglycemia, chronic kidney disease, obesity, and hyperhomocysteinemia (HHcy), promotes atherosclerosis development via several mechanisms. These mechanisms include endothelial cell (EC) activation and injury (Shao et al., 2014; Yin et al., 2015; Li et al., 2016a, 2018a); monocyte recruitment and differentiation (Combadière et al., 2008; Fang et al., 2014); decreased function of regulatory T cells (Tregs) (Ait-Oufella et al., 2006; Yan et al., 2008; Li et al., 2017); transdifferentiation of Tregs into antigen-presenting cell (APC) like Tregs (Xu et al., 2018); impaired vascular repairability of bone marrow-derived progenitor cells (Du et al., 2012; Barbier et al., 2015; Li et al., 2016b); increased migration and proliferation of vascular smooth muscle cells (Monroy et al., 2015; Ferrer et al., 2016), and high fat-induced adipocyte hypertrophy and metabolic healthy obesity (Virtue et al., 2017). Human aortic endothelial cell (HAEC) activation is widely understood to contribute substantively to the early development of atherosclerosis by increasing leukocyte recruitment through upregulation of adhesion molecules such as ICAM1 (Tabas et al., 2015; Vestweber, 2015; Gimbrone and García-Cardeña, 2016). We have recently reported that exposure to proatherogenic stimuli, such as the proinflammatory lysophospholipid lysophosphatidylcholine (LPC), can induce prolonged HAEC activation as part of early atherogenesis (Li et al., 2016a,c, 2018b); this phenomenon is one of a larger class of conditional danger-associated molecular patterns (DAMPs) that trigger the onset of oxidative, autoimmune, and inflammatory tissue damages (Wang X. et al., 2016; Shao et al., 2017). However, the regulatory mechanisms underpinning these physiological changes remain poorly understood; and whether and how non-coding RNAs (ncRNAs) mechanisms control HAEC activation remains poorly characterized.

For many years, proteins have represented the primary functional end product of genetic information, though the genes that encode them account for less than 2% of the genome (Anastasiadou et al., 2018). As new sequencing technology advances, as many as six types of ncRNAs have been identified, including three small ncRNA types [i.e., RNA interference (RNAi), tRNA-derived stress-induced RNA (tiRNA), and small nuclear RNA (snRNA)] and three long RNAs types [i.e., transfer RNA (tRNA), ribosomal RNA (rRNA), and long non-coding RNAs (lncRNA)] (Gomes et al., 2018). RNAi can be categorized into five subgroups including small interfering RNA (siRNA), piwi-interacting RNA (piRNA), and microRNA (miRNA), repeat associated siRNA (rasiRNA), and agotron. LncRNAs can be further classified into six subtypes: sense lncRNA, intronic lncRNA, antisense lncRNA, intergenic lncRNA, enhancer RNA (eRNA), and circular RNA (circRNA). These ncRNAs together constitute almost 60% of the transcriptional output in human cells, and they have been shown to regulate cellular processes and pathways in developmental and pathological contexts through mRNA degradation or modification, transcription regulation, translation regulation, and miRNA sponging, among other functions (Mattick and Makunin, 2006; Bartel, 2009; Cech and Steitz, 2014; Lasda and Parker, 2014). We previously reported that a group of anti-atherogenic miRNAs might suppress proatherogenic gene expression (Virtue et al., 2011, 2012; Mai et al., 2012). We also found that microRNA-155 (miR155) is among several miRs significantly upregulated in atherogenic apolipoprotein E deficient (ApoE-/-) mouse aortas. MiR155 promotes HAEC activation and mouse atherosclerosis, whereas the deficiency of miR155 in ApoE-/- background leads to high fat-induced adipocyte hypertrophy and establishes a novel metabolically healthy obese (MHO) mouse model (Virtue et al., 2017). Among 109 miRNAs that we examined in all four hyperlipidemia-related diseases (HRDs), namely atherosclerosis, non-alcoholic fatty liver disease (NAFLD), obesity and type II diabetes (T2DM), miR-155 and miR-221 are significantly modulated in these HRDs. MiR-155 is significantly upregulated in atherosclerosis and decreased in other HRDs. MiR-221 is increased in three HRDs but reduced in obesity. These findings led to our new classification of types I and II MHOs, which are regulated by miR-221 and miR-155, respectively. We also found that the proinflammatory adipokine, resistin, is significantly increased in white adipose tissues (WAT) of the MHO mice, revealing our newly proposed, miR-155-suppressed “secondary wave inflammatory state (SWIS),” characteristic of MHO transition to classical obesity (CO) (Johnson et al., 2018). Our and others’ results demonstrate that ncRNAs play significant roles in regulating the pathogenesis and progression of cardiovascular diseases and other diseases.

**FIGURE 1:**
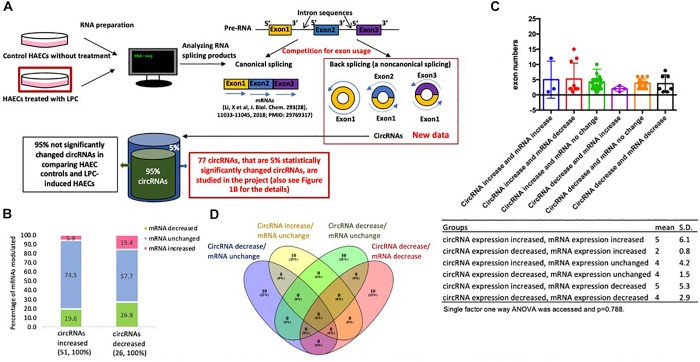
**(A)** Lysophosphatidylcholine (LPC)-induced top 5% significantly changed circular RNAs (circRNAs) are studied in human aortic endothelial cells (HAECs) in comparison to that of control HAECS. This is a flow chart to outline how we analyzed the 5% top significantly changed circular RNAs from LPC-treated HAECs in comparison to that from control HAECs. **(B)** The majority (48 out of 51,94.1%) of circRNA expression increases while related mRNA expressions remain constant or decrease. **(C)** There is no significant differences in exon numbers among circRNA groups. **(D)** There are no overlaps among the top 10 pathways in the following four groups.

Two types of lncRNAs (i.e., linear and circular forms) have been found in the lncRNA family. In contrast to previous models, recent work has shown that circRNAs are relatively abundant and may be the predominant transcripts of hundreds of genes. The spliceosome machinery produces circRNAs by direct back-splicing of exons or introns within pre-mRNA or by exon skipping. As a result of a direct back-splicing event, an exon is not associated with an adjacent downstream exon, as seen in linear splicing, but with an upstream exon or intron. This back-splicing gives rise to circular RNA molecules harboring exons or introns that are out-of-order from the genomic context (Figure 1A). These circular molecules are ubiquitously expressed in human and more than 30,000 different circRNAs have been identified. They are regulated in several disease states and contribute to disease development and progression (Gomes et al., 2018). Although the importance of circRNAs in regulating EC function has been reported, the comprehensive analyses of circRNAs in EC activation, EC function, and cardiovascular diseases remain at their infancy; additionally, the characterization of circRNAs in atherosclerosis-relevant HAECs has not been reported (Maass et al., 2017; Huang et al., 2018; Shang et al., 2018).

Regardless of the progress, we identified several important knowledge gaps to be filled in this study. We hypothesized that circRNAs might play an important role in mediating homeostatic status in HAECs activated by proatherogenic LPC. We analyzed our RNA-Seq raw data sets, obtained from control-, and LPC- treated HAECs (Li et al., 2018b), using two well-accepted circular RNA databases such as CircExplorer2 and CIRI to obtain a list of significantly changed circular RNAs (circRNAs) (Gao et al., 2015; Zhang et al., 2016) 4. We found that this list of 77 circRNAs may play homeostatic roles in activated HAECs via novel mechanisms including exon usage competition, miRNA sponging (Hansen et al., 2013; Memczak et al., 2013) and potential generation of short proteins for protein interaction disruption. We also found that novel mechanisms, such as (a) flanking intron homology, (b) LPC-induced modulation of spliceosome components, and (c) upstream chromatin long-range interaction, play important roles in promoting the generation of circRNAs in LPC-activated HAECs. These novel insights may lead to the identification of new therapeutic targets for treating metabolic cardiovascular diseases, inflammations, and cancers.

## Materials and Methods

### Generation of CircRNA Datasets

Raw RNA-Seq data was obtained from a previous study (Li et al., 2018b). Raw reads generated by the Illumina HiSeq control software were assessed using FastQC (Andrews, 2010). Samples with poor sequence quality were discarded. Sequence reads were mapped to the hg38 genome using STAR (Dobin et al., 2013). Fusion junction reads were parsed using CIRCexplorer2 and captured back-splicing junction reads were then annotated with UCSC annotation files (Zhang et al., 2016). For additional detections, sequence reads were mapped to the hg38 genome using the Burrows-Wheeler Alignment tool (Li and Durbin, 2009), circRNAs were identified using CIRI (Gao et al., 2015). Gene annotation was performed using org.Hs.eg.db (Carlson, 2017). A list of circRNAs common to both control and LPC-stimulated HAECs was generated using the VLOOKUP function in Microsoft Excel. To filter out significantly changed circRNAs, ratios of control to LPC-stimulated circRNA read numbers were calculated, and base 2 logarithms taken of these ratios. Mean and standard deviation of logarithm values were calculated using MATLAB and used to generate a 95% confidence interval (mean ± 2^∗^SD). Using previously obtained mRNA expression data (Li et al., 2018b), an mRNA fold change confidence interval was similarly calculated using the ten housekeeping genes: C1orf43, CHMP2A, GAPDH, EMC7, GPI, PSMB2, PSMB4, RAB7A, SNRPD3, and VPS29; and it was used to separate circRNAs by corresponding mRNA expression change.

### Comparison of Flanking Intron Sequences

The generated circRNA data included flanking intron coordinates; a Python script was used to parse these coordinates and feed them into an NIH NCBI BLAST+ command line query (blastn -max_hsps 1 -outfmt “10 pident *e*-Value bitscore”) comparing the sequences of 3′ and 5′ flanking intron sequences using local copies of hg38 human genome primary assembly chromosome sequences^1^ (Camacho et al., 2009). This returned percent identity, the expected value (number of matches of equal strength expected by chance), and bitscore values (normalized score for match strength) for each pair of flanking introns. These were then exported to a text file and imported into a Microsoft Excel spreadsheet for tabulation. Bitscore values were further used for statistical analysis (described below under “Statistical Analysis”).

### Matching CircRNAs to Database Entries

The genomic length of each significantly changed circRNA was calculated by subtracting its genomic start coordinate from its genomic end coordinate. A MATLAB script was then used to find circRNAs with matching length and gene locus from a local copy of the circRNAdb database and to export all results to a Microsoft Excel spreadsheet (Schliebs et al., 1996; Chen et al., 2016). Notably, genomic coordinates in circRNAdb were given with respect to the older hg37 human genomic annotation. A local copy of the hg37 genomic annotation was therefore obtained for comparison^2^. Then, NIH NCBI BLAST+ queries between original hg38 coordinates and CircRNAdb hg37 coordinates (blastn -max_hsps 1 -outfmt “10 pident”) were performed using a MATLAB script (Schliebs et al., 1996; Camacho et al., 2009), taking only the single best match for each pair of sequences, the percent identity values of which confirmed sequence alignment for all length matches found. CircInteractome also uses the hg37 human genome annotation but a different set of IDs; matches in CircInteractome were determined by hand due to incomplete downloadable data sets.

### Analysis of Long–Range Interactions

A complete list of long–range chromatin interaction sites in the human genome was obtained from the 4DGenome database^3^ as a tabulated text file (Teng et al., 2015). The grep command line utility was used to filter for entries detected using Hi-C methodology and involving at least one circRNA-related gene. The resulting filtered data was imported into Microsoft Excel and raw interaction distances calculated as the differences between gene start coordinates. An AWK script was used to determine whether the circRNA-related gene was downstream or upstream of its partner in each interaction pair and to add this information to the data file. The signs of distance values were then updated, with downstream entries designated as positive and upstream values designated as negative, using a Python script. These updated distance values were separated into the same six groups as their corresponding circRNA-related genes and used in pairwise two-sample Kolmogorov–Smirnov tests (described further below under “Statistical Analysis”). Distance distributions for all upregulated and all downregulated circRNAs were compared by groups overall as well as by only upregulated and downregulated circRNAs with increased, unchanged, or decreased corresponding mRNA expression, respectively.

### Characterizing Open Reading Frame Sequences

Open reading frame (ORF) peptide sequences and internal ribosomal entry site (IRES) data were obtained to identify significantly changed circRNAs from circRNAdb (Chen et al., 2016). A MATLAB script was used to call remote NIH NCBI BLAST+ peptide alignments for all ORF sequences against the NCBI non-redundant protein sequences database (nr^4^) (Schliebs et al., 1996; Camacho et al., 2009), restricted to entries for *Homo sapiens* (blastp -db nr -remote -entrez_query “Homo sapiens [Organism]” -max_target_seqs 1 -outfmt “10 sacc pident *e*-Value”). This returned the subject accession, percent identity, and expected value of the single best database match, which was then exported with the gene name and circRNAdb ID to a Microsoft Excel spreadsheet. The obtained accessions were individually and manually searched on the NCBI protein database^5^ to determine if they corresponded to canonical mRNA transcripts from the same gene locus as the corresponding circRNA. A modified version of the hg38/hg37 comparison MATLAB script was also used to run NIH NCBI BLAST+ searches for the Kozak consensus sequence gccRccAUGG in genomic sequences of all significantly changed circRNAs (Kozak, 1987; Schliebs et al., 1996; Camacho et al., 2009).

### Pathway Analysis

QIAGEN Ingenuity Pathway Analysis (IPA) software, which constructs predicted upstream and downstream causal networks for input datasets from a curated research literature base, was used to elucidate potential downstream pathways for sponged miRNAs (Krämer et al., 2014). Lists of miRNAs were input into IPA and ran through the miRNA target filter to generate a list of potential mRNA targets. The list of mRNA target genes was then run through an IPA core analysis. All canonical downstream pathways returned in the resulting output were exported into a Microsoft Excel spreadsheet. The top ten pathways were extracted for further qualitative consideration.

### Graphical Figure Generation

For three-group Venn diagrams, lists of genes were input into an online Venn diagram generator (^6^Evolutionary Genomics, Ghent University, Gent, Belgium) (Draw Venn Diagram). This tool was used to produce both diagrams and lists of overlapped genes between groups. For six-group Venn diagrams, gene lists were input into the InteractiVenn online tool^7^ to generate diagrams (Heberle et al., 2015), while the above Ghent University tool was again used to produce lists of overlapped genes between groups (Draw Venn Diagram). Explanatory and conceptual graphics were produced using Microsoft Paint.

### Statistical Analysis

Descriptive summary statistics were reported by group. Data were checked for normality assumption and, if found to be not normally distributed, subsequently transformed using various functions such as the log10 and cubic-root to find the optimal transformation for the underlying chromatin long-range interaction distance distribution data. Chromatin long-range interaction distance density functions were then estimated and plotted by group under the optimal transformation using the non-parametric kernel density approach with a normal weight function (Jones et al., 1996; Hollander and Wolfe, 1999; Silverman, 2018). Pairwise comparisons of median chromatin long-range interaction distance among the six groups were performed with multiple comparison adjustments using the Dwass, Steel, and Critchlow-Fligner method based on the Wilcoxon test for downstream and upstream data separately (Douglas and Michael, 1991; Conover, 1999; Hollander and Wolfe, 1999; Shalabh, 2011). Pairwise comparisons between groups for chromatin long-range interaction distance distribution, median distance location shift, and distance distribution scale were implemented using the Kolmogorov–Smirnov two-sample test, Hodges–Lehmann estimation method and Fligner-Policello test, and Ansari-Bradley test, respectively, again for downstream and upstream data separately (Lehmann, 1963; Douglas and Michael, 1991; Conover, 1999; Hollander and Wolfe, 1999; Shalabh, 2011). SAS version 9.4 was used to perform these analyses and generate density function plots for the chromatin long-range interaction distance data.

For rest of the data, single-factor ANOVA and non-parametric Kruskal–Wallis tests were conducted for all multi-group analyses using the Real Statistics Resource Pack add-in for Microsoft Excel^8^ (Zaiontz, 2013). For confidence intervals, MATLAB was used to calculate mean and standard deviations for data sets (Schliebs et al., 1996). Probabilities of ratios of upregulation to downregulation were calculated by summing binomial coefficients and dividing by the appropriate power of 2 in MATLAB (Schliebs et al., 1996), as given by the formula $p=\frac{\sum_{i=n}^{u}\left(\begin{array}{c} i\\ u \end{array}\right)}{2^{u}}$, where *u* is the total number of genes considered and *n* is the large number of upregulated or downregulated genes.

## Results

### LPC Modulates the Expression of a List of 77 Circular RNAs in HAECs; and the Majority of Which, 94.1% of Increased CircRNAs, Compete With Related mRNAs for Exon Usage

Our preliminary data processing detected 3,170 distinct circRNAs in control cells and 3,486 circRNAs in a proatherogenic lipid LPC-treated HAECs as we reported (Li et al., 2016a). To better analyze our data set, we derived a list of 1,093 circRNAs found in both the control and LPC-treated data sets before calculating read number ratio – the quotient of the read number in the LPC-treated data set and the corresponding read number in the control data set – for each circRNA in the list to serve as a measure of expression change (Figure 1A). We then took the base-2 logarithm of each readnumber ratio (log ratio) and calculated a 95 percent confidence interval (-0.3815 ± 2.3395) for log ratio, taking all the 77 circRNAs with log ratios outside this confidence interval to have significantly changed levels of expression in LPC-treated HAECs (Table 1A). Then, we separated these circRNAs into two groups of increased and decreased circRNA expression. To assess potential relationships between circRNAs and their corresponding mRNAs published, we then further divided each of these groups into three subgroups based on increased, unchanged, or decreased expression of corresponding mRNA transcripts, with all fold change levels within a 95 percent confidence interval (1.01 ± 0.1) determined by the housekeeping genes C1orf43, CHMP2A, GAPDH, EMC7, GPI, PSMB2, PSMB4, RAB7A, SNRPD3, and VPS29 considered to indicate unchanged mRNA expression (Table 1B), thereby obtaining six groups of significantly changed circRNAs classified by changes in both circRNA and corresponding mRNA expression.

**Table 1A:** LPC-induced 77 circRNAs in HAECs can be classified into 6 groups based on expression change of circRNAs and corresponding (related) mRNAs.

Gene ID	Gene	circRNAdb ID	Readnumber (LPC)	Readnumber (Control)	Ratio	Log(2) ratio	FC (mRNA)	Index (circRNA exons)	Exon count	Exon count (genomic)
circRNA expression increased, mRNA expression increased (Shao et al., 2014)											
23094	SIPA1L3	hsa_circ_10577	9	2	4.5	2.17	1.12	11,12	2	24
117583	PARD3B	N/A	4	1	4	2	1.19	4,5,6,7,8,9,10,11, 12,13,14,15	12	33
116840	CNTROB	hsa_circ_09218	4	1	4	2	1.11	13	1	22
circRNA expression decreased, mRNA expression increased (Li et al., 2016a)											
2530	FUT8	hsa_circ_02544	1	7	0.14	–2.81	1.2	3	1	15
768211	RELL1	hsa_circ_08846	2	15	0.13	–2.91	1.15	6,5,4	3	8
51150	SDF4	hsa_circ_21644	1	9	0.11	–3.17	1.15	4,3	2	8
64778	FNDC3B	hsa_circ_15231	1	15	0.07	–3.91	1.13	5,6	2	30
circRNA expression increased, mRNA expression unchanged (Huang et al., 2018)											
55852	TEX2	hsa_circ_31354	15	1	15	3.91	0.98	7,6,5	3	15
5820	PVT1	hsa_circ_16350	8	1	8	3		2	1	9
51232	CRIM1	hsa_circ_16396	15	2	7.5	2.91	1.03	2,3,4	3	22
1793	DOCK1	hsa_circ_00689	7	1	7	2.81	1.05	2,3,4,5,6,7,8,9,10,11, 12,13,14,15,16,17, 18,19,20,21,22,23	22	56
4670	HNRNPM	hsa_circ_20744	7	1	7	2.81	1.01	2,3,4,5	4	16
22937	SCAP	hsa_circ_19380	7	1	7	2.81	0.94	8,7,6,5,4	5	25
9341	VAMP3	hsa_circ_18051	7	1	7	2.81	0.94	3,4	2	5
N/A	RP11-146B14.1	N/A	6	1	6	2.58	#N/A	12,13,14,15	4	
9380	GRHPR	hsa_circ_03935	6	1	6	2.58	1.06	2,3,4	3	11
27086	FOXP1	hsa_circ_19469	6	1	6	2.58	1.01	12,11,10,9,8	5	32
64750	SMURF2	hsa_circ_01208	5	1	5	2.32	1.11	10,9,8,7,6	5	19
84456	L3MBTL3	N/A	5	1	5	2.32	1.03	4,5,6,7,8,9,10,11, 12,13,14,15,16, 17,18,19,20,21	18	30
862	RUNX1T1	N/A	5	1	5	2.32	0.99	5,4,3,2	4	20
151050	KANSL1L	hsa_circ_06859	5	1	5	2.32	0.96	4,3,2	3	23
11138	TBC1D8	hsa_circ_32540	5	1	5	2.32	0.94	15,14,13,12,11,10	6	25
84328	LZIC	hsa_circ_31394	5	1	5	2.32	0.94	4,3,2	3	10
23331	TTC28	hsa_circ_22261	5	1	5	2.32	0.93	3,2	2	29
1456	CSNK1G3	hsa_circ_08140	14	3	4.67	2.22	0.93	2,3,4	3	18
8460	TPST1	hsa_circ_22617	9	2	4.5	2.17	0.98	2,3	2	8
#N/A	PRKY	hsa_circ_30664	4	1	4	2	N/A	4,5,6,7	4	8
#N/A	PCNXL2	hsa_circ_10712	4	1	4	2	N/A	23,22	2	
1523	CUX1	hsa_circ_12403	8	2	4	2	1.11	21	1	34
9701	PPP6R2	hsa_circ_07544	4	1	4	2	1.11	2,3	2	29
54919	DNAAF5	N/A	4	1	4	2	1.1	9,10,11	3	14
84293	FAM213A	N/A	4	1	4	2	1.06	2,3,4	3	9
7813	EVI5	hsa_circ_11187	4	1	4	2	1.04	19,18,17	3	30
81608	FIP1L1	hsa_circ_18069	4	1	4	2	1.02	11,12,13	3	19
51295	ECSIT	hsa_circ_00599	4	1	4	2	1	4,3	2	9
152002	XXYLT1	hsa_circ_02143	4	1	4	2	1	3,2	2	11
6310	ATXN1	hsa_circ_13397	4	1	4	2	0.99	8	1	11
1024	CDK8	hsa_circ_27078	4	1	4	2	0.98	10,11,12	3	14
6259	RYK	hsa_circ_26048	4	1	4	2	0.98	13,12,11,10,9,8,7	7	15
92	ACVR2A	hsa_circ_04751	4	1	4	2	0.98	2,3,4	3	12
22828	SCAF8	hsa_circ_06913	4	1	4	2	0.98	2,3,4,5,6	5	22
29086	BABAM1	hsa_circ_04303	4	1	4	2	0.96	7,8	2	9
23244	PDS5A	hsa_circ_29001	4	1	4	2	0.95	21,20,19,18,17	5	36
7443	VRK1	hsa_circ_08594	4	1	4	2	0.94	2,3,4,5,6,7,8,9,10,11	10	15
8826	IQGAP1	hsa_circ_10932	4	1	4	2	0.93	6,7,8,9	4	39
circRNA expression decreased, mRNA expression unchanged (Li et al., 2016b)											
23362	PSD3	hsa_circ_02851	1	7	0.14	–2.81	1.05	8,7,6,5	4	29
55833	UBAP2	hsa_circ_30156	1	7	0.14	–2.81	0.94	12,11,10,9	4	30
10443	N4BP2L2	hsa_circ_12240	1	7	0.14	–2.81	0.93	6,5,4,3	4	22
6904	TBCD	hsa_circ_00754	2	15	0.13	–2.91	1.05	14,15,16,17,18,19	6	61
5747	PTK2	hsa_circ_23485	2	15	0.13	–2.91	1.03	5,4,3	3	44
23603	CORO1C	hsa_circ_01619	1	9	0.11	–3.17	1.06	8,7	2	20
5917	RARS	hsa_circ_21209	1	9	0.11	–3.17	1.01	2,3,4,5	4	15
11174	ADAMTS6	hsa_circ_28538	3	29	0.1	–3.27	1.05	7,6,5,4,3,2	6	33
22955	SCMH1	hsa_circ_12377	1	10	0.1	–3.32	1.09	10,9	2	22
51306	FAM13B	hsa_circ_11413	1	10	0.1	–3.32	1.01	10,9,8	3	30
2050	EPHB4	hsa_circ_08510	1	10	0.1	–3.32	1	12,11	2	17
444	ASPH	hsa_circ_10828	1	11	0.09	–3.46	1	3,2	2	33
57488	ESYT2	hsa_circ_25060	1	11	0.09	–3.46	0.95	13,12,11,10,9	5	26
10905	MAN1A2	hsa_circ_02643	1	15	0.07	–3.91	1.06	2,3,4,5	4	14
50807	ASAP1	hsa_circ_09642	1	15	0.07	–3.91	0.93	14,13,12,11,10,9	6	37
circRNA expression increased, mRNA expression decreased (Yan et al., 2008)											
4649	MYO9A	N/A	8	1	8	3	0.75	39,38,37,36,35,34, 33,32,31,30,29,28	12	48
10564	ARFGEF2	N/A	5	1	5	2.32	0.91	5,6,7	3	39
51444	RNF138	hsa_circ_01057	5	1	5	2.32	0.9	3,4	2	8
152006	RNF38	hsa_circ_32756	5	1	5	2.32	0.79	4,3	2	19
23047	PDS5B	hsa_circ_01546	4	1	4	2	0.91	2,3	2	38
55719	SLF2	N/A	4	1	4	2	0.9	5,6	2	21
55247	NEIL3	hsa_circ_00262	4	1	4	2	0.84	8,9	2	10
56886	UGGT1	N/A	4	1	4	2	0.78	2,3,4,5,6,7,8,9,10, 11,12,13,14,15,16	15	44
23499	MACF1	N/A	4	1	4	2	0.68	42,43,44,45,46,47, 48,49,50,51,52	11	111
11123	RCAN3	hsa_circ_22201	4	1	4	2	0.67	2	1	7
circRNA expression decreased, mRNA expression decreased (Combadière et al., 2008)											
659	BMPR2	hsa_circ_06837	1	7	0.14	–2.81	0.84	2,3	2	13
23253	ANKRD12	hsa_circ_31497	1	7	0.14	–2.81	0.78	2,3,4,5,6,7	6	18
10725	NFAT5	hsa_circ_16704	1	8	0.13	–3	0.64	15	1	19
26057	ANKRD17	hsa_circ_01636	1	9	0.11	–3.17	0.91	29	1	37
55125	CEP192	hsa_circ_26363	1	9	0.11	–3.17	0.85	2,3,4,5,6,7,8,9	8	46
121512	FGD4	hsa_circ_21030	1	9	0.11	–3.17	0.85	5,6,7,8,9,10	6	27
22836	RHOBTB3	hsa_circ_19124	1	17	0.06	–4.09	0.91	6,7	2	14


**Table 1B:** LysoPC does not significantly modulate the expressions of housekeeping mRNAs in human aortic endothelial cells (HAECs), suggesting that the RNA-Seq experiments in HAECs treated with lysoPC were well performed.

Housekeeping genes	Fold change

C1orf43	0.97
CHMP2A	1
GADPH	1
EMC7	0.98
GPI	1
PSMB2	0.98
PSMB4	1.02
RAB7A	0.98
SNRPD3	1
VPS29	1.04


We also matched each circRNA, if possible, with its corresponding ID in the circRNA database CircRNAdb (Chen et al., 2016), to provide for more consistent identification, and for later analysis of the database’s open reading frame data. Of note, among 51 circRNAs increased, 48 out of 51 related mRNAs (94.1%) are either unchanged (38 out of 51, 74.5%) or decreased (10 out of 51, 19.5%), while only three increased both mRNA and circRNA expression (5.9%, Figure 1B). Similarly, among 26 circRNAs decreased, 19 out of 26 related mRNAs (73.1%) are either unchanged (15 out of 26, 57.7%) or increased (4 out of 26, 15.4%), while only seven decreased both mRNA and circRNA expression (26.9%, Figure 1B). The results suggest that a dominant inversely correlative relationship between circRNA expression and mRNA expression levels potentially indicates exon usage competition during splicing for either circRNA expression or mRNA expression.

We also compared the average number of exons per circRNA transcript of each group using single-factor ANOVA to assess any potential impact of circRNA length on expression patterns, but no significant differences were found (*p* = 0.788) (Figure 1C). Considering the slightly right-skewed distributions of exon numbers, we also performed a Kruskal–Wallis test of the same data, which also failed to indicate significance (*p* = 0.46309). These results suggest that transcript length has little to no role in modulating circRNA expression following LPC treatment in HAECs. Taken together, our results suggest that the proatherogenic lipid LPC induces a list of circRNAs in HAECs; and that a dominant inversely correlative relationship between circRNA and mRNA expression levels potentially indicates exon usage competition during splicing.

### CircRNA-Related Genes Do Not Share Any Top Pathways With LPC-Induced mRNA Pathways; and Genes Associated With Significantly Changed CircRNAs Exhibit Little Relationship With Potential Effects of Inflammation-Related Cytokine Stimulation

To determine the signaling pathways associated with circRNA-related genes, we performed the IPA. As shown in Table 1C, we found the following: *first*, six top pathways related to the unchanged genes which related circRNAs were increased, including pyridoxal 5′-phosphate salvage pathway, Fcγ receptor-mediated phagocytosis in macrophage and monocyte, transforming growth factor-β (TGF-β) signaling, salvage pathways of pyrimidine ribonucleotides, epithelial adherens junction signaling, and Wnt/β-catenin signaling; *second*, ten top pathways related to the unchanged mRNAs which related circRNAs were decreased, including ephrin B signaling, focal adhesion kinase (FAK) signaling, axonal guidance signaling, ephrin receptor signaling, integrin signaling, interleukin-15 (IL-15) production, tRNA charging, semaphoring signaling in neurons, ephrin A signaling, and regulation of cellular mechanics by calpain protease; *third*, ten top pathways related to the unchanged RNAs which related circRNAs were increased, including pyridoxal 5′-phosphate salvage pathway, Fcγ receptor-mediated phagocytosis, TGF-β signaling, salvage pathways of pyrimidine ribonucleotides, epithelial adherens junction signaling, Wnt/β-catenin signaling, actin cytoskeleton signaling, cell cycle control of chromosomal replication, unfolded protein response, remodeling of epithelial adherens junctions; and *fourth*, ten top pathways related to the decreased mRNAs which related circRNAs were decreased, including role of osteoblasts, osteoclasts, and chondrocytes in rheumatoid arthritis, neuroinflammation signaling pathway, cardiomyocyte differentiation via bone morphogenetic protein (BMP) receptors, April mediated signaling, B cell activation factor signaling, Wnt/Ca++ pathway, netrin signaling, BMP signaling pathway, regulation of IL-2 expression in activated and anergic T lymphocytes and factors promotion of cardiogenesis in vertebrates. The Vann Diagram analysis found that no signaling pathways are overlapped among the four groups (Figure 1D). The results suggest that, although we cannot determine directly circRNAs functional pathways in this LPC-induced HAEC activation, LPC-induced mRNAs-related HAEC activation pathways, that we reported (Li et al., 2018b), have no overlaps with any signaling pathways for circRNAs-related mRNAs.

**Table 1C:** There are no overlaps among the top 10 pathways in the following four groups #, suggesting that the functions of circRNAs-related mRNAs do not participate in LPC-induced human aortic endothelial cell activation.

Number	LPC upregulated mRNAs (promoting endothelial activation, PMID)	CircRNA increased and mRNA unchanged genes (inflammation, PMID)
1	Superpathway of cholesterol biosynthesis (HAEC activation, 29371247)	Pyridoxal 5′-phosphate salvage pathway (neurological pathologies, 22201923)
2	Cholesterol biosynthesis I	Fcγ Receptor-mediated phagocytosis in macrophages and monocytes (immune responses, 28117787)
3	Cholesterol biosynthesis II (via 24,25-dihydrolanosterol)	TGF-β signaling (anti-inflammation, 22438968)
4	Cholesterol biosynthesis III (via Desmosterol)	Salvage pathways of pyrimidine ribonucleotides (repair and inflammatory signal, 28053879)
5	Granulocyte adhesion and diapedesis	Epithelial adherens junction signaling (epithelial cell migration, 30010460)
6	Hepatic fibrosis/hepatic stellate cell activation	Wnt/β-catenin signaling (immune response, 25136145)
7	Role of macrophages, fibroblasts, and endothelial Cells in rheumatoid arthritis	Actin cytoskeleton signaling (immune response, 30274167)
8	Role of IL-17F in allergic inflammatory airway diseases	Cell cycle control of chromosomal replication (immune response, 29908233)
9	Agranulocyte adhesion and diapedesis	Unfolded protein response (inflammatory response, 30278941)
10	p38 MAPK signaling	Remodeling of epithelial adherens junctions (epithelial cell migration, 30010460)

**Number**	**CircRNA decreased and mRNA unchanged genes**	**CircRNA decreased and mRNA decreased genes**

1	Ephrin B signaling	Role of osteoblasts, osteoclasts, and chondrocytes in rheumatoid arthritis
2	FAK signaling	Neuroinflammation signaling pathway
3	Axonal guidance signaling	Cardiomyocyte differentiation via BMP receptors
4	Ephrin receptor signaling	April mediated signaling
5	Integrin signaling	B Cell activating factor signaling
6	IL-15 production	Wnt/Ca+ pathway
7	tRNA charging	Netrin signaling
8	Semaphorin signaling in neurons	BMP signaling pathway
9	Ephrin A signaling	Regulation of IL-2 expression in activated and anergic T lymphocytes
10	Regulation of cellular mechanics by calpain protease	Factors promoting cardiogenesis in vertebrates


*CircRNAs may play roles in weakening the roles of related mRNAs via their roles in exon usage competition. #Of note, there were no top ten pathways identified by the Ingenuity Pathway Analysis in three other groups in Table 1A.*

We further hypothesized that LPC-induced circRNA expression could be positively associated with changes in gene expression under other pro-inflammatory stimuli. Thus, to compare the effects of LPC stimulation with that of prototypical inflammation-related responses, we obtained mRNA expression change data for cells stimulated with the pro-inflammatory cytokines tumor necrosis factor alpha (TNF-α), interferon gamma (IFN-γ), and interleukin 6 (IL-6) and the anti-inflammatory cytokines TGF-β, IL-4, and IL-10 from the database ArrayExpress (Braun et al., 2013; Teles et al., 2013; Bald et al., 2014; Jin et al., 2014; Kharma et al., 2014; Montoya et al., 2014; Polak et al., 2014, 2017; Duncan et al., 2015; Kolesnikov et al., 2015; Tan et al., 2015). We found all circRNA-related genes significantly changed after stimulation in each data set (not shown) and results indicate that cytokines with similar effects did not tend to regulate circRNA-related genes in similar directions, nor did pro-inflammatory and anti-inflammatory cytokines tend to regulate circRNA-related genes in opposing directions (not shown). This in turn suggests a limited or absent canonical role for circRNA-related genes in inflammatory response and fails to indicate general trends for pro- or anti-inflammatory cytokine modulation of circRNA-related genes. However, it neither supports nor disproves the roles of inflammatory signaling in regulating circRNA levels, as circRNA levels are largely independent of the mRNA expression levels used in this analysis and, furthermore, circRNAs may fulfill different physiological functions such as homeostasis than their canonically spliced mRNA counterparts do such as promotion of HAEC activation induced by LPC.

### 68.8% of the CircRNAs Feature Significant Flanking Intron Homology

**FIGURE 2:**
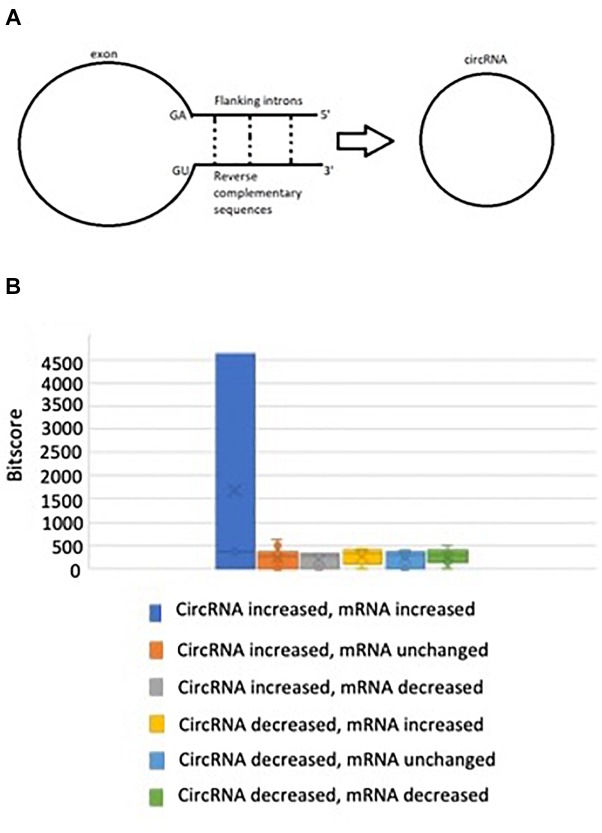
**(A)** Pre-splicing RNA transcripts with reverse complementary matches in flanking intron sequences tend to favor circRNA biogenesis, **(B)** There is no significant difference in bitscore between groups when non-parametric testing is performed.

To identify the potential mechanism underlying the generation of circRNAs, previous reports found that many circRNA transcripts feature reverse complementary flanking intron sequences, which enable self-pairing that brings 3′ and 5′ exonic sequences into close proximity, an arrangement that favors head-to-tail alternative splicing and circRNA biogenesis (Figure 2A; Albury et al., 2015). To confirm this pattern in our dataset, we ran NIH NCBI BLAST sequence alignments using NIH NCBI BLAST+ command line tools (blastn -max_hsps 1 -outfmt “10 pident *e*-Value bitscore”) comparing the 3′ and 5′ flanking intron sequences for each circRNA, as previously described (Albury et al., 2015). We found that 53 out of 77 circRNAs (68.8%) possessed flanking intron sequences with significant reverse complements as determined by expected value (number of matches expected given random sequences) <1^∗^10^-20^ (Table 2). This number is lower than that of human 88% circRNA with the Alu repeats in flanking regions (Albury et al., 2015), and that in C. elegans circRNAs (96.3%); and high than that of Zebrafish circRNAs (2.5%) (Ivanov et al., 2015). To test potential association of reverse complementarity of flanking introns with different expression patterns of circRNA and mRNA, we performed a single-factor ANOVA using the bitscores (normalized values reporting strength of alignment between sequences) of circRNAs from each of the six groups sorted previously, assigning a bitscore of zero (indicating no matches better than those resulting from chance) for all circRNAs whose flanking introns did not demonstrate reverse complementarity. This initial analysis indicated significant differences between groups (*p* = 0.0011), but further inspection revealed the presence of an outstanding data point (bitscore = 4617) in the “circRNA increased, mRNA increased” group, which combined with the small size (*n* = 3) of the group greatly weakened the generalizability of the test (Figure 2B). We thus performed a non-parametric Kruskal–Wallis test on the same data set, but this failed to reach significance (*p* = 0.74053). We also performed another single-factor ANOVA with the outstanding point removed, the results of which marginally failed to indicate any significant difference in bitscore between groups (*p* = 0.07877). Thus, we cannot conclude that there is an association between flanking intron homology and circRNA expression patterns, although that our statistical analysis approaches significance suggests that the area may warrant further study, especially with a larger sample. Our results indeed demonstrate that most circRNAs feature significant flanking intron homology, suggesting that this mechanism may be underlying selection for the expressions of circRNAs versus mRNAs.

**Table 2:** 53 out of 77 (68.8%) circRNAs have significant homologies between flanking introns, which may facilitate chromatin looping and circRNA generation.

Gene	circRNAdb ID	% identity	*e*-Value	Bitscore
circRNA expression increased, mRNA expression increased				
PARD3B	N/A	98.44	0.00	4617
SIPA1L3	hsa_circ_10577	90.24	0.00	374
circRNA expression decreased, mRNA expression increased				
FUT8	hsa_circ_02544	93.17	0.00	429
RELL1	hsa_circ_08846	88.03	0.00	335
FNDC3B	hsa_circ_15231	84.59	0.00	311
circRNA expression increased, mRNA expression unchanged				
PCNXL2	hsa_circ_10712	98.65	0.00	525
RUNX1T1	hsa_circ_02162	89.74	0.00	427
DOCK1	hsa_circ_00689	89.61	0.00	387
TTC28	hsa_circ_22261	89.6	0.00	375
ATXN1	hsa_circ_13397	89.45	0.00	300
XXYLT1	hsa_circ_02143	89.26	0.00	521
ECSIT	hsa_circ_00599	88.63	0.00	361
CUX1	hsa_circ_12403	87.25	0.00	333
EVI5	hsa_circ_11187	86.53	0.00	363
SMURF2	hsa_circ_01208	86.23	0.00	324
IQGAP1	hsa_circ_10932	86.13	0.00	291
TEX2	hsa_circ_31354	85.26	0.00	316
TBC1D8	hsa_circ_32540	84.04	0.00	263
TPST1	hsa_circ_22617	83.83	0.00	560
LZIC	hsa_circ_31394	83.55	0.00	278
SCAF8	hsa_circ_06913	83.05	0.00	261
KANSL1L	hsa_circ_06859	82.95	0.00	191
VRK1	hsa_circ_08594	82.95	0.00	115
DNAAF5	N/A	82.87	0.00	254
PDS5A	hsa_circ_29001	82.68	0.00	257
PPP6R2	hsa_circ_07544	82.34	0.00	499
ACVR2A	hsa_circ_04751	81.88	0.00	255
VAMP3	hsa_circ_18051	81.76	0.00	250
PRKY	hsa_circ_30664	81.66	0.00	230
CSNK1G3	hsa_circ_08140	80.89	0.00	226
PVT1	hsa_circ_16350	76.23	0.00	619
circRNA expression decreased, mRNA expression unchanged				
FAM13B	hsa_circ_11413	89.9	0.00	379
SCMH1	hsa_circ_12377	89.74	0.00	387
N4BP2L2	hsa_circ_12240	88.45	0.00	363
ADAMTS6	hsa_circ_28538	88.41	0.00	353
PSD3	hsa_circ_02851	87.66	0.00	351
UBAP2	hsa_circ_30156	87.63	0.00	326
PTK2	hsa_circ_23485	85.49	0.00	396
ASAP1	hsa_circ_09642	84.95	0.00	294
MAN1A2	hsa_circ_02643	84.03	0.00	272
EPHB4	hsa_circ_08510	82.08	0.00	257
circRNA expression increased, mRNA expression decreased				
UGGT1	N/A	87.2	0.00	326
RNF38	hsa_circ_32756	86.81	0.00	318
RNF138	hsa_circ_01057	86.3	0.00	315
SLF2	N/A	85.76	0.00	315
RCAN3	hsa_circ_22201	83.84	0.00	278
PDS5B	hsa_circ_01546	82.06	0.00	254
circRNA expression decreased, mRNA expression decreased				
RHOBTB3	hsa_circ_19124	90.64	0.00	392
ANKRD12	hsa_circ_31497	85.86	0.00	313
ANKRD17	hsa_circ_01636	85.37	0.00	296
CEP192	hsa_circ_26363	84.76	0.00	307
BMPR2	hsa_circ_06837	81.99	0.00	503
FGD4	hsa_circ_21030	78.65	0.00	115


### LPC Stimulation Disrupts the Expression Levels of 17 Spliceosome Components, Potentially Promoting Back Splicing for CircRNA Formation Over Canonical Splicing for mRNAs

**FIGURE 3:**
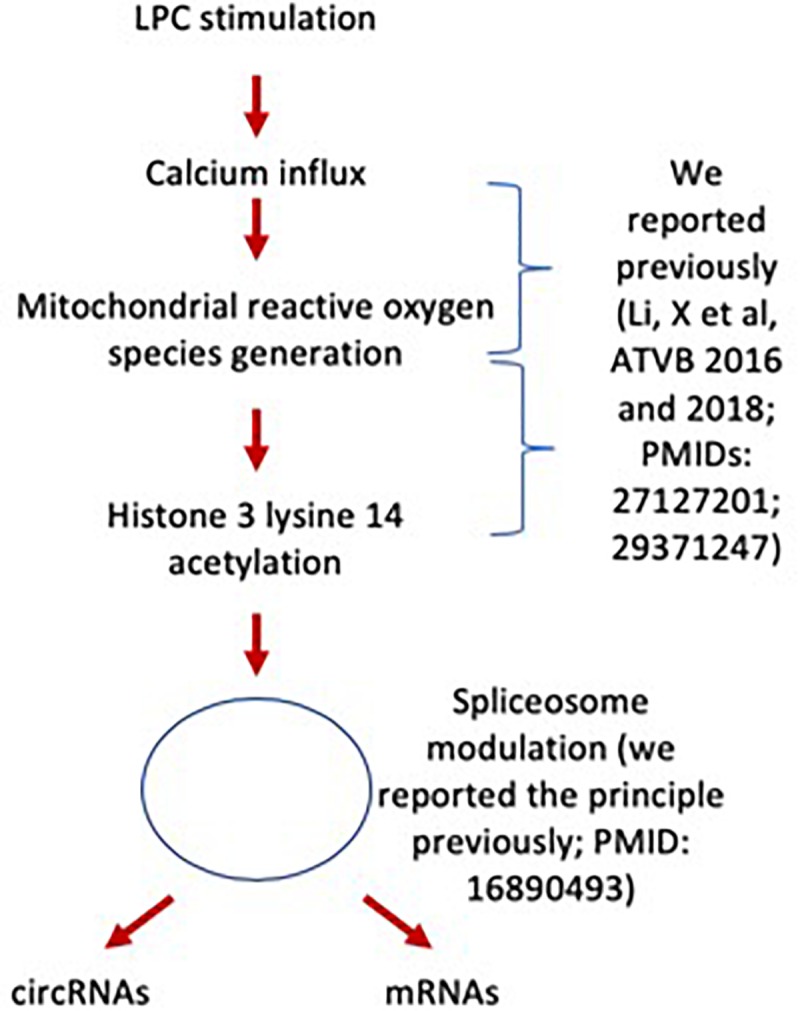
LPC modulates the expressions of spliceosome components and may facilitate the generation of circRNAs.

We and others reported that downregulation of certain spliceosome components promotes circRNA formation over canonical mRNA splicing (Shen et al., 2017; Wilusz, 2018); and that proinflammatory cytokine TNF-a, inflammation (Liang et al., 2017), autoimmune disease (Xiong et al., 2006b), and tumors modulate alternative splicing (Ng et al., 2004) and spliceosome (Yan et al., 2004). To determine whether this potential mechanism plays any roles in shaping LPC-modulated circRNA expression patterns, we compared the list of significantly changed mRNAs from RNA-Seq (determined via housekeeping gene confidence interval, as previously described) with lists of known spliceosome proteins (Deckert et al., 2006; Yang et al., 2006; Behzadnia et al., 2007; Bessonov et al., 2008; Table 3). Six spliceosome components were significantly downregulated after LPC stimulation, while another 11 were significantly upregulated. Given that 51 (66.2%) circRNAs were significantly upregulated while only 26 (33.8%) were significantly downregulated (*p* = 0.0029, where *p* represents the probability of a ratio equal to or more extreme than the experimental value, assuming circRNA upregulation and downregulation equally likely) and that among upregulated circRNAs, 10 corresponding mRNAs experienced significant downregulation while only 3 experienced significant upregulation (*p* = 0.0461, where *p* represents the probability of a ratio equal to or more extreme than the experimental value, assuming mRNA upregulation and downregulation equally likely) (Figure 1A and Table 3), this disruption may have been sufficient to shift the splicing patterns from canonical mRNA splicing toward back splicing for circRNA formation (Figure 3), as reported previously (Wilusz, 2018). While the data currently available is insufficient to establish a causal link between LPC-induced spliceosome disruption and a shift from canonical splicing to circRNA formation, it does demonstrate a strong association suitable for more intensive study. Taken together, our results suggest that LPC stimulation disrupts the expression levels of 17 spliceosome components, potentially promoting back splicing for circRNA formation over canonical splicing for mRNAs.

**Table 3:** Seventeen spliceosome genes out of approximately 80 proteins have fold changes outside a housekeeping gene-determined 95 percent confidence interval in RNA-Seq data.

Gene	FC
FUS	0.68
PCBP2	0.68
RBM7	0.77
THOC1	0.82
LSM8	0.83
PCBP1	0.87
RBM10	1.11
EIF4A3	1.12
CCDC130	1.12
RBM42	1.13
DGCR14	1.13
MFAP1	1.18
PUF60	1.18
THOC3	1.20
LSM6	1.21
RNF113A	1.24
FAM50B	1.25


*There are 6 protein downregulation and 11 proteins upregulation.*

### Shifting Chromatin Long–Range Interaction Sites From Downstream to Upstream Is Associated With Induction of a List of Circular RNAs in LysoPC-Activated Human Aortic Endothelial Cells; and in Contrary, Shifting Chromatin Long–Range Interaction Sites From Upstream to Downstream Is Associated With Induction of a List of mRNAs

**FIGURE 4:**
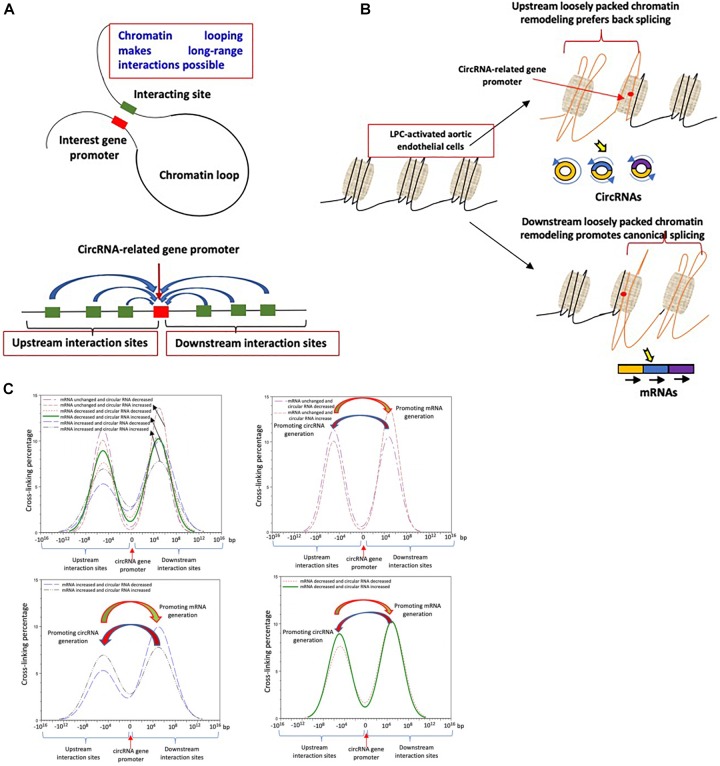
**(A)** Chromatin long range interaction may regulate the expressions of circRNAs-related genes via forming chromatin loops. **(B)** Loosely packed chromatin regions either upstream or downstream from circRNAs related gene promoters may differentially prefer back splicing to generate circRNAs to canonical splicing to generate mRNAs. **(C)** Shifting chromatin long-range interaction sites from downstream to upstream may promotes a list of circular RNAs; and in contrast, shifting chromatin long-range interaction sites from upstream to downstream promotes induction of a list of mRNAs. The data were collected from the experimental data deposited in the 4DGenome database (https://4dgenome.research.chop.edu) containing the data from human umbilical vein endothelial cells (HUVECs).

Rapid development of technologies in capturing chromosome conformations such as chromosome conformation capture sequencing (3C-Seq) (Wahl et al., 2009), chromosome conformation capture-on-chip (4C-Seq) (Dekker et al., 2002; Splinter et al., 2011), and chromosome conformation capture carbon copy sequencing (5C-Seq) (Stadhouders et al., 2013) allows determination of the relative frequency of direct physical contact between a pair of linearly separated chromatin segments with 3C-Seq, and genome–wide interactions involving a single anchor region, and interactions involving multiple genomic regions with 4C-Seq and 5C-Seq, respectively (Figure 4A). Differences in chromatin long-range interaction patterns between genes have previously been hypothesized to influence alternative splicing (Dostie et al., 2006) and the transcription of inflammatory genes such as cytokine (Acuña and Kornblihtt, 2014) and cytokine receptor (Park et al., 2016) and cardiovascular disease causative genes (Deligianni and Spilianakis, 2012). We hypothesize that chromatin long–range interactions differentially regulate the gene promoters to differentiate the mRNA increase from circRNA increase versus decrease, derived from the same gene, under LPC stimulation in HAECs. To test this hypothesis with respect to circRNA biogenesis, we obtained long-range interaction data for all genes related to significantly changed circRNAs from the well-accepted 4DGenome database with a huge collection of 4,433,071 experimentally derived chromatin long–range interactions (Teng et al., 2015). We then calculated the distances between interacting genes with respect to the circRNA-related gene, with distances designated as positive if the circRNA-related gene was localized downstream of its partner and as negative if the circRNA-related gene was localized upstream of its partner. The two-sample Kolmogorov–Smirnov test of the chromatin long-range interaction distance between genes corresponding to downregulated and upregulated circRNAs indicated a significant difference between the two distance distributions (*p* < 0.001). *First*, the majority of long-range interaction sites with circRNA-related genes were focused on 10^6^ kb upstream and downstream of the circRNA-related gene, suggesting a common feature of the long-range chromatin interaction sites with LPC-induced circRNA-related genes; *Second*, in the three groups with circRNA related genes such as (a) upregulation; (b) downregulation; or (c) no changes induced by LPC, the group with LPC-induced upregulation of mRNAs had the lowest peak, the group with LPC-induced downregulation of mRNAs had the middle peak, the group with LPC-induced no changed expression of mRNAs had the highest peak, suggesting that high concentration of chromatin long-range interaction sites prevents HAECs from responding to LPC stimulation in changing gene expressions (Figure 4B,C); *Third*, in both groups of mRNA upregulation and mRNA downregulation induced by LPC, circRNA increase was associated with increased upstream chromatin long-range interaction sites, suggesting that an increase in upstream chromatin long-range interaction sites is associated with circRNA generation presumably by favoring back-splicing in generating circRNAs over canonical splicing in generating mRNAs (Figure 4C). In contrast, a decrease in upstream chromatin long-range interaction sites is associated with mRNA generation presumably by favoring canonical splicing in generating mRNAs over back-splicing in generating circRNAs (Figure 4C). Altogether, these results suggest that different long-range interaction mechanisms are likely to govern circRNA upregulation and downregulation. Indeed, upregulated circRNAs tended to interact at somewhat longer distances than downregulated circRNAs did (Figure 4B,C). Since the 4DGenome database contains the experimental data derived from human non-aortic ECs (Teng et al., 2015), the future work will be needed to use circular chromosome conformation capture sequencing (4C-Seq) to examine HAECs stimulated with LPC to map the specific upstream interaction sites for a favorable selection of circRNA over mRNAs. Therefore, our results may suggest that certain ideal long-range interaction distances exist that are more likely to promote circularization; and that favorable distances tend to be greater in magnitude than unfavorable ones.

### Six Significantly Changed CircRNAs Sponge miRNAs, Which May Be Associated With Inhibition of Type 2 Diabetes Mellitus, Inhibition of Migration, and Inhibition of Vascular Dysfunction and Immune Responses in Endothelial Cells

**FIGURE 5:**
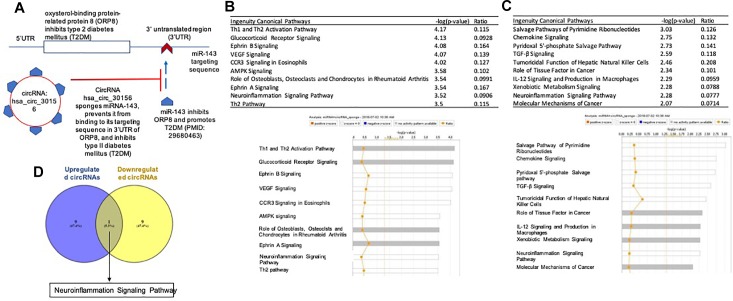
**(A)** CircRNA has the potential role to inhibit type 2 diabetes mellitus (T2DM)by sponging miRl43 and preventing miRl43 from binding to oxysterol-binding protein-related protein 8 (ORP8) ˆuntranslated region (3′UTR). **(B)** Upregulated circRNAs sponge miRNAs involved in both pro- and anti-inflammatory pathways. **(C)** Downregulated circRNAs sponge miRNAs are involved with both pro-inflammatory and anti-inflammatory pathways. **(D)** One pathway is overlapped in the sponged microRNAs between upregulated and downregulated circRNAs.

A number of circRNAs can sponge miRNAs, preventing miRNA-mediated degradation of mRNA transcripts and disruption of canonical signaling and effector pathways (Hansen et al., 2013; Memczak et al., 2013). Thus, by regulating miRNA activity, significantly changed circRNAs can act through canonical pathways, potentially playing a significant role in HAEC activation. We examined the sponging potential of all significantly changed circRNAs using the CircInteractome database (Montefiori et al., 2018), recording two miRNAs with four or more predicted binding sites in a single circRNA transcript, a threshold above which meaningful sponging activity is likely to occur Memczak et al. (2013). Another four significantly changed circRNAs are experimentally shown to sponge miRNAs (Dudekula et al., 2016; Chen et al., 2017; Yan et al., 2017; Wang et al., 2018), for six total circRNAs with miRNA sponging activity including miR125, miR143, miR1272, miR153, miR515-5p, and miR196a-5p (Table 4). In Figure 5A, to demonstrate the proof of principle, we showed how circRNA has_circ_30156 could inhibit type 2 diabetes mellitus (T2DM) by sponging miR143 and preventing miR143 from binding to oxysterol-binding protein-related protein 8 (ORP8) 3′untranslated region (3′UTR).

**Table 4:** Six circRNAs demonstrate miRNA sponging activities.

Gene	circRNAdb ID	miRNA sponged	Readnumber (LPC)	Readnumber (control)	Ratio	Function of microRNA	PMID
PVT1	hsa_circ_16350	miR-125	8	1	8	Promote regeneration/repair	29461585
UBAP2	hsa_circ_30156	miR-143	1	7	0.14	Inhibit autophage/promote inflammation	29562274
VAMP3	hsa_circ_18051	miR-1272	7	1	7	T cell differentiation	26941729
VRK1	hsa_circ_08594	miR-153	4	1	4	Inhibit migration/invasion	29113423
FGD4	hsa_circ_21030	miR-515-5p	1	9	0.11	Decrease in atopic eczema	17622355
DOCK1	hsa_circ_00689	miR-196a-5p	7	1	7	Delay lymph node metastasis	29434944


To elucidate potential downstream pathways for these miRNAs that might influence the LPC-induced inflammatory response, we separately performed IPA for miRNAs sponged by upregulated and downregulated circRNAs, respectively. Notably, the top two pathways for miRNAs sponged by upregulated circRNAs were the pro-inflammatory type 1 T helper cell (Th1), and Th2 activation pathway, and the anti-inflammatory glucocorticoid receptor signaling pathway (Figure 5B). Similarly, miRNAs sponged by downregulated circRNAs were prominently implicated in the anti-inflammatory transforming growth factor-β (TGF-β) signaling pathway and the pro-inflammatory interleukin-12 (IL-12) signaling and production in macrophages pathway (Figure 5C). Owing to these numerous conflicting results, the pro- and anti-inflammatory effects of the predicted downstream pathways likely cancel in large part, resulting in little overall pro- or anti-inflammatory effects (Figure 5D). Specifically, microRNA-125a plays crucial roles both in development and in the adult tissues, including it: (1) contributes to the control of phase transitions in development and/or cell differentiation; (2) regulates the expression of several target proteins that are involved in cell proliferation, apoptosis, and migration; (3) interferes with the expression of the hepatitis B virus surface antigen in liver cells, thus counteracting viral replication (Zhang et al., 2017). miR-125b plays a significant role in immune response and apoptosis (Potenza and Russo, 2013). The novel long intergenic non-coding RNA UCC promotes colorectal cancer progression by sponging miR-143 (Ribeiro and Sousa, 2014). The function of miR1272 remains unreported. miR153 is tumor suppressive (Huang et al., 2017), and miR153 targeting of KCNQ4 contributes to vascular dysfunction in hypertension (Zhao et al., 2013). miR-515-5p controls cancer cell migration through MARK4 regulation (Carr et al., 2016). Higher expression of miR-196a was both associated with poor overall survival and disease-free survival and recurrence-free survival in different kinds of cancers (Pardo et al., 2016). Taken together, sponging of most of six miRs in ECs may be associated with inhibition of migration, inhibition of vascular dysfunction and immune responses.

### 74% Significantly Changed CircRNAs Contain Open Reading Frames, the Majority of Which Encode Fragments of Known Proteins

**FIGURE 6:**
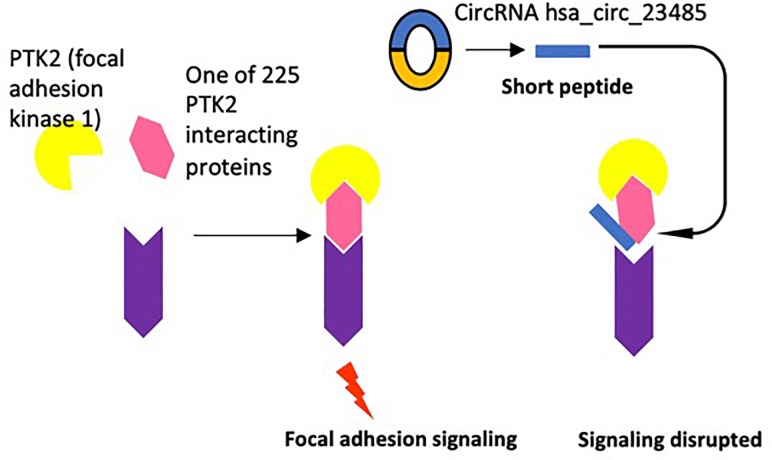
One example of potential disruption of protein–protein interactions by circRNA encoded short peptides is demonstrated.

Despite their classification as ncRNAs, numerous circRNAs can be translated into peptides, some of which are novel forms with uncharacterized yet potentially relevant function (Cai et al., 2016; Legnini et al., 2017; Pamudurti et al., 2017). Moreover, the circRNAdb database used to assign circRNA IDs provides ORF peptide sequence data for all circRNAs with ORFs (Chen et al., 2016). To assess the potential of significantly changed circRNAs to encode small proteins/peptides, we performed NIH NCBI BLAST peptide sequence alignments against the NCBI non-redundant protein sequences database (nr), restricted to entries for *Homo sapiens*, for all significantly changed circRNAs with ORF data in circRNAdb using the NIH NCBI BLAST+ command line utility (blastp -db nr -remote -entrez_query “Homo sapiens [Organism]” -max_target_seqs 1 -outfmt “10 sacc pident *e*-Value”) (Table 5A). Out of 77 significantly changed circRNAs, 57 (74.0%) contained ORFs; of these, 48 ORF peptide sequences matched substantially (with an expected value < 1^∗^10^-4^) to fragments of known proteins. Notably, 47 of the 48 ORFs with substantial matches mapped to a protein encoded by mRNA corresponding to the same gene as the circRNA, the sole exception originating from a circRNA corresponding to the non-coding gene PVT1. A majority of significantly changed circRNAs, therefore, have protein-coding potential and could produce fragments of known proteins. Such protein fragments remain both uncharacterized and experimentally undetected, but could theoretically serve physiological functions, including ones different from those of the full canonical protein (Figure 6). Furthermore, using predicted IRES data from circRNAdb, we found that all 57 circRNAs with ORFs contain at least one predicted IRES, and 32 of them (56.1%) contain at least one predicted IRES with an *R*-value (a metric indicating suitability of a sequence as an IRES) greater than a previously determined cutoff value of 1.54 and a predicted pseudoknot structure, which together indicate a greater probability of ribosomal entry and subsequent translation (Yang et al., 2017). We previously reported that this type of IRES drive secondary open reading frames is translated into short proteins (Wu et al., 2009); and that short peptides can disrupt the protein–protein interactions (Xiong et al., 2006a). The Kozak consensus sequence is a sequence which occurs on eukaryotic mRNA and has the consensus (gcc) gccRccAUGG, which plays a major role in the initiation of the translation process (Yang et al., 2005). However, NIH NCBI BLAST searches for Kozak (1996) sequences insignificantly changed circRNAs did not yield any matches.

**Table 5A:** All but one circRNA open reading frames with significant BLAST matches encode peptides from the same gene as their corresponding mRNAs.

Gene	circRNAdb ID	Accession	% ID	*E*-value	Same translation as mRNA?
Near-perfect overlap with known sequences				
VAMP3	hsa_circ_18051	XP_016858330	100	0.00	Yes
DOCK1	hsa_circ_00689	Q14185	100	0.00	Yes
HNRNPM	hsa_circ_20744	BAG57075	100	0.00	Yes
FOXP1	hsa_circ_19469	BAC11065	100	0.00	Yes
TBC1D8	hsa_circ_32540	BAA76517	100	0.00	Yes
KANSL1L	hsa_circ_06859	XP_011509011	100	0.00	Yes
RUNX1T1	hsa_circ_02162	AAB34820	100	0.00	Yes
CSNK1G3	hsa_circ_08140	XP_016864559	100	0.00	Yes
TPST1	hsa_circ_22617	XP_011514936	100	0.00	Yes
RCAN3	hsa_circ_22201	NP_001238914	100	0.00	Yes
NEIL3	hsa_circ_00262	Q8TAT5	100	0.00	Yes
PPP6R2	hsa_circ_07544	EAW73534	100	0.00	Yes
VRK1	hsa_circ_08594	2LAV_A	100	0.00	Yes
BABAM1	hsa_circ_04303	BAG61451	100	0.00	Yes
RYK	hsa_circ_26048	AAH21700	100	0.00	Yes
ATXN1	hsa_circ_13397	NP_000323	100	0.00	Yes
ACVR2A	hsa_circ_04751	AAH67417	100	0.00	Yes
PRKY	hsa_circ_30664	O43930	100	0.00	Yes
UBAP2	hsa_circ_30156	BAG64527	100	0.00	Yes
RELL1	hsa_circ_08846	XP_016864079	100	0.00	Yes
NFAT5	hsa_circ_16704	XP_016878362	100	0.00	Yes
CEP192	hsa_circ_26363	XP_016881293	100	0.00	Yes
SDF4	hsa_circ_21644	XP_011539858	100	0.00	Yes
CORO1C	hsa_circ_01619	BAH14436	100	0.00	Yes
ADAMTS6	hsa_circ_28538	XP_011541429	100	0.00	Yes
ASAP1	hsa_circ_09642	CAD97831	100	0.00	Yes
MAN1A2	hsa_circ_02643	XP_016855604	100	0.00	Yes
ANKRD17	hsa_circ_01636	XP_016863500	99.796	0.00	Yes
ANKRD12	hsa_circ_31497	BAB15014	99.683	0.00	Yes
CRIM1	hsa_circ_16396	XP_016859748	99.448	0.00	Yes
PSD3	hsa_circ_02851	XP_016868754	99.333	0.00	Yes
PDS5B	hsa_circ_01546	AAH70274	99.048	0.00	Yes
FNDC3B	hsa_circ_15231	EAW78473	98.864	0.00	Yes
FUT8	hsa_circ_02544	BAA92858	98.551	0.00	Yes
PCNXL2	hsa_circ_10712	BAG51507	98.387	0.00	Yes
RHOBTB3	hsa_circ_19124	EAW96050	98.148	0.00	Yes
PTK2	hsa_circ_23485	NP_001339677	98.052	0.00	Yes
SCAP	hsa_circ_19380	EAW64826	97.5	0.00	Yes
EPHB4	hsa_circ_08510	4AW5_A	97.5	0.00	Yes
IQGAP1	hsa_circ_10932	AAH05906	97.458	0.00	Yes
XXYLT1	hsa_circ_02143	XP_005269343	97.403	0.00	Yes
SMURF2	hsa_circ_01208	EAW94194	97.03	0.00	Yes
CNTROB	hsa_circ_09218	XP_016879630	97.015	0.00	Yes
LZIC	hsa_circ_31394	NP_001303904	96.97	0.00	Yes
TTC28	hsa_circ_22261	XP_011528324	96.947	0.00	Yes
PVT1	hsa_circ_16350	EAW92103	95.918	0.00	No (non-coding gene)
FAM13B	hsa_circ_11413	XP_016865040	87.786	0.00	Yes
N4BP2L2	hsa_circ_12240	EAX08513	78.261	0.00	Yes (fragment of same gene)
Partial overlap with known sequences				
BMPR2	hsa_circ_06837	NP_057675	56	3.10	
ESYT2	hsa_circ_25060	EAW95833	48	3.20	
GRHPR	hsa_circ_03935	EAW60274	39.286	3.00	
No overlap with known sequences				
RNF38	hsa_circ_32756				
SIPA1L3	hsa_circ_10577				
SCAF8	hsa_circ_06913				
CDK8	hsa_circ_27078				
ECSIT	hsa_circ_00599				
FGD4	hsa_circ_21030				


We previously reported that short peptides with high homology to the interaction domain sequences of protein interaction partners could interfere the protein interaction (Yang et al., 2005). To demonstrate the proof of principle, in Figure 6, as suggested by the experimental data in the NIH-NCBI-Gene database^9^, focal adhesion kinase 1 (PTK2) has as many as 225 interaction proteins (Table 5B and Figure 6), thus, the circRNA hsa_circ_23485 encoded short peptide(s) could disrupt the protein interaction between PTK2 and interaction proteins. Taken together, 74% significantly changed circRNAs contain open reading frames, the majority of which encode fragments of known proteins, which may interfere with protein interaction-based signaling pathways. Of note, in addition to perform experiments in the future to verify the circRNAs, we also notice that various novel computational methods for finding ncRNAs versus coding RNAs (Abbas et al., 2016) and identifying small peptides (Pauli et al., 2015) have been developed, so that we will collaborate in the future with bioinformatics experts to verify the generation of those potential short peptides in HAECs.

**Table 5B:** 75 of 77 circRNA-related genes have numerous interactions with other proteins, suggesting that functional interactions between the circRNAs-related proteins and their interaction proteins may be disrupted by circRNA-encoded short peptides.

Gene name	circRNAdb ID	Interactions
TEX2	hsa_circ_31354	7
MYO9A	N/A	27
PVT1	hsa_circ_16350	2
CRIM1	hsa_circ_16396	3
VAMP3	hsa_circ_18051	74
DOCK1	hsa_circ_00689	37
SCAP	hsa_circ_19380	29
HNRNPM	hsa_circ_20744	341
GRHPR	hsa_circ_03935	34
FOXP1	hsa_circ_19469	47
RP11-146B14.1	N/A	N/A
RNF38	hsa_circ_32756	17
SMURF2	hsa_circ_01208	201
ARFGEF2	N/A	53
RNF138	hsa_circ_01057	45
TTC28	hsa_circ_22261	25
LZIC	hsa_circ_31394	13
L3MBTL3	N/A	38
TBC1D8	hsa_circ_32540	6
KANSL1L	hsa_circ_06859	0
RUNX1T1	hsa_circ_02162	96
CSNK1G3	hsa_circ_08140	38
SIPA1L3	hsa_circ_10577	39
TPST1	hsa_circ_22617	13
MACF1	N/A	85
RCAN3	hsa_circ_22201	14
UGGT1	N/A	65
PARD3B	N/A	7
NEIL3	hsa_circ_00262	19
CNTROB	hsa_circ_09218	123
SLF2	N/A	9
PDS5B	hsa_circ_01546	54
CUX1	hsa_circ_12403	69
DNAAF5	N/A	66
IQGAP1	hsa_circ_10932	294
PPP6R2	hsa_circ_07544	69
VRK1	hsa_circ_08594	34
PDS5A	hsa_circ_29001	76
BABAM1	hsa_circ_04303	67
FAM213A	N/A	23
EVI5	hsa_circ_11187	18
SCAF8	hsa_circ_06913	22
RYK	hsa_circ_26048	93
FIP1L1	hsa_circ_18069	81
CDK8	hsa_circ_27078	120
ATXN1	hsa_circ_13397	416
ACVR2A	hsa_circ_04751	48
ECSIT	hsa_circ_00599	117
XXYLT1	hsa_circ_02143	10
PRKY	hsa_circ_30664	8
PCNXL2	hsa_circ_10712	3
ANKRD12	hsa_circ_31497	17
BMPR2	hsa_circ_06837	99
N4BP2L2	hsa_circ_12240	29
UBAP2	hsa_circ_30156	34
PSD3	hsa_circ_02851	8
FUT8	hsa_circ_02544	30
RELL1	hsa_circ_08846	19
TBCD	hsa_circ_00754	64
PTK2	hsa_circ_23485	225
NFAT5	hsa_circ_16704	10
FGD4	hsa_circ_21030	5
CEP192	hsa_circ_26363	78
SDF4	hsa_circ_21644	92
ANKRD17	hsa_circ_01636	40
CORO1C	hsa_circ_01619	109
RARS	hsa_circ_21209	146
ADAMTS6	hsa_circ_28538	2
SCMH1	hsa_circ_12377	41
FAM13B	hsa_circ_11413	6
EPHB4	hsa_circ_08510	30
ESYT2	hsa_circ_25060	34
ASPH	hsa_circ_10828	44
FNDC3B	hsa_circ_15231	12
ASAP1	hsa_circ_09642	44
MAN1A2	hsa_circ_02643	17
RHOBTB3	hsa_circ_19124	35


## Discussion

We previously published experimental data outlining the role of LysoPC (LPC) on HAEC (Li et al., 2018c). In this previous publication we demonstrated that LysoPC stimulation leads to: (1) the upregulation of inflammatory adhesion molecules in HAEC; (2) upregulation of cytokine transcript expression; and (3) promotion of prolonged HAEC activation and expression of innate immune co-stimulation molecule expression. We believe that this RNA-Seq (RNA sequencing) data analyses provide strong experimental evidence for the role of LysoPC stimulation of proinflammatory mRNAs. Given that alternative splicing events lead to the generation of circRNAs from mRNA precursors in other cell types (Wilusz, 2018), there is strong justification to explore the role of LysoPC-induced circRNAs.

Although the importance of circRNAs in regulating EC function has been reported, the comprehensive analyses of circRNAs in EC activation, EC function, and cardiovascular diseases remain at their infancy; and the characterization of circRNAs in atherosclerosis-relevant HAECs has not been reported (Maass et al., 2017; Huang et al., 2018; Shang et al., 2018). To fill in this important knowledge gap, we profiled the circRNAs in HAECs stimulated with we proposed “conditional danger associated molecular pattern” LPC (Wang X. et al., 2016), and compared the circRNAs significantly modulated in HAECs by LPC with the mRNAs modulated in the same sets of RNA-Seq samples (Li et al., 2018b).

In the current study, we made the following findings: *first*, LPC modulates the expression of a list of 77 circRNAs in HAECs; and the majority of which, 94.1% of increased circRNAs, compete with related mRNAs for exon usage; *second*, circRNAs-related genes do not share any top pathways with LPC-induced mRNA pathways, and genes associated with significantly changed circRNAs exhibit little relationship with potential effects of inflammation-related cytokine stimulation; *third*, 68.8% of the circRNAs feature significant flanking intron homology; *fourth*, LPC stimulation disrupts the expression levels of 17 spliceosome components, potentially promoting back splicing for circRNA formation over canonical splicing for mRNAs; *fifth*, shifting chromatin long-range interaction sites from downstream to upstream promotes induction of a list of circRNAs in lysoPC-activated HAECs; and in contrary, shifting chromatin long-range interaction sites from upstream to downstream promotes induction of a list of mRNAs; *sixth*, six significantly changed circRNAs sponge miRNAs, which may be associated with inhibition of type 2 diabetes mellitus, inhibition of migration, inhibition of vascular dysfunction and immune responses in ECs; and *seventh*, 74% significantly changed circRNAs contain open reading frames, the majority of which encode fragments of known proteins, suggesting that short proteins generated from the secondary open reading frames potentially driven by internal ribosome entry sites may interfere the protein-protein interaction-based signaling.

As reported previously, LPC stimulation causes significant changes in mRNA expression levels for many genes (Li et al., 2018b). IPA of these changed genes implicates several common upstream regulatory signaling pathways, the most significant of which are the sterol regulatory element binding transcription factor 2 (SREBF2), SREBF chaperone (SCAP), and SREBF1 pathways (not shown), which jointly serve as key lipid metabolism regulators and contribute to pro-inflammatory signaling (Shao and Espenshade, 2012). This establishes a novel association between upregulated pro-inflammatory lipid metabolism pathways and significant expression changes for 77 circRNAs. Our data are insufficient to conclude any roles of lipid metabolism pathways in circRNA expression modulation. However, given the strength of the association between the significantly changed upstream pathways and the significantly changed circRNAs, more direct future investigation of the effects of lipid metabolism pathway disruption on circRNA levels will be appropriate and could confirm or extend this observation.

It is highly unlikely that the extreme ratio of upregulated to downregulated circRNAs or that of upregulated to downregulated corresponding mRNAs among upregulated circRNAs under LPC stimulation would occur by chance, as noted earlier. For this reason, spliceosome depletion, which is experimentally demonstrated to cause such responses in circRNA and mRNA expression (Liang et al., 2017; Wilusz, 2018), continues to be a compelling area for future study despite the inconclusive results presented in this study. For instance, knockdown of downregulated or overexpression of upregulated spliceosome components identified after LPC stimulation, potentially combined with more robust circRNA detection methods such as qPCR verification of back-spliced sequences (Jeck and Sharpless, 2014; Szabo and Salzman, 2016), could clarify the role of each component in LPC-induced disruption of circRNA expression patterns.

Additionally, the dramatic expression changes of many circRNAs under LPC stimulation suggests that they play currently undetermined, possibly significant roles in HAEC activation and the proatherogenic response. Indeed, the circRNA *circular antisense non-coding RNA in the INK4 locus* (circANRIL) performs anti-atherogenic, anti-proliferative functions *in vitro* by inhibiting ribosomal biogenesis through assembly factor binding (Holdt et al., 2016), although this circRNA was not detected in our data set. Nevertheless, it is possible that some significantly changed circRNAs fulfill regulatory roles in atherogenesis through similar mechanisms. As circRNAs are better characterized and prediction of RNA-protein binding becomes more sophisticated, it may be possible to detect protein-binding functions, including those related to atherogenesis or atheroprotection, in our significantly changed circRNAs.

Likewise, many of our significantly changed circRNAs could encode proteins. Although the ORFs detected match the loci of canonically spliced mRNAs, many of the peptides encoded are likely to be fragments of canonical proteins and therefore may serve different functions. Knock-in experiments of these novel predicted peptides could uncover new insights about their potential roles in LPC-induced atherogenesis. Furthermore, as our current findings do not include protein expression data, additional assay experiments under LPC stimulation, such as Western blots and high-performance liquid chromatography-linked mass spectrometer analyses specific to these novel predicted peptides, would provide additional insight on their expression patterns. Given the established coding potential of circRNAs (Legnini et al., 2017; Pamudurti et al., 2017; Yang et al., 2017, such microproteins remain an underexplored pathway for circRNA regulatory roles in atherogenesis.

Furthermore, the field of circRNAs is still in its infancy. Software detection methods may give widely differing results, and not all circRNAs predicted from RNA-Seq data can be verified experimentally through methods such as RNase R-catalyzed degradation (Hansen et al., 2016). Simply repeating an LPC-induced HAEC activation experiment, treating with RNase R to remove spurious linear transcripts, and using additional circRNA algorithms to corroborate findings could provide more accurate data on circRNA expression change; using qPCR confirmation for particularly meaningful or significant detections would likewise further improve data reliability. Additionally, recent studies have outlined a number of novel experimental methods for verifying the expression and function (Jeck and Sharpless, 2014; Petkovic and Muller, 2015; Barrett and Salzman, 2016; Werfel et al., 2016; Liu et al., 2017; Zeng et al., 2017; Gomes et al., 2018; Li et al., 2018b). Overall, however, changes in circRNA expression levels following LPC stimulation follow unusual and poorly understood patterns, and further, more intensive profiling under otherwise identical conditions could reveal regulatory pathways not apparent in this study, which uses relatively simple detection methods. To this end, future studies are required using clinical arterial samples from atherosclerotic patients and healthy controls through the collaboration with vascular surgeons using the Temple University Institutional Review Board approved protocol.

**FIGURE 7:**
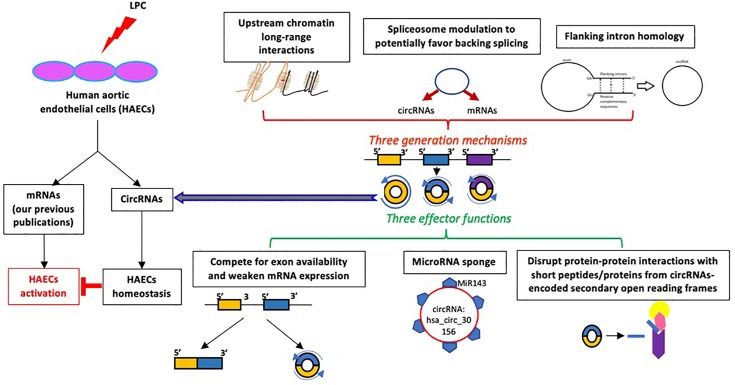
A new working model: Proatherogenic lipid, LPC, induces the upregulation of a group of circular RNAs in human aortic endothelial cells, which may be generated by three mechanisms and play homeostatic functions via three effector mechanisms.

Taken together, based on our findings, we propose a novel working model (Figure 7): *first*, modulation of 77 significantly changed circRNAs in LPC-activated HAECs is temporally associated with LPC-upregulated cholesterol synthesis-SREBP2 pathway and LPC-downregulated TGF-β pathway that we reported recently (Li et al., 2018b); *second*, the generation of circRNAs in LPC-activated HAECs may be facilitated by three novel molecular mechanisms including: (i) high flanking intron homologies, (ii) LPC-induced modulation of spliceosome components, and (iii) increased upstream chromatin long–range interactions; and *third*, circRNAs in LPC-activated HAECs may play homeostatic functions by acting on a few novel aspects, including: S (a) competition with related mRNAs for exon usage, (b) miRNA sponge, and (c) secondary open reading frame-generated short peptide disruption of protein-protein interaction. We acknowledge that circRNAs profiling data have brought a huge amount of new findings for detailed experimental verifications in the future. Nevertheless, these novel insights may lead to identification of new therapeutic targets for treating metabolic cardiovascular diseases, inflammations, and cancers.

## Author Contributions

AL and YS carried out the data gathering, data analysis, and prepared the tables and figures. CD, YL, DY, YZ, XL, CJ, CY, WY, KM, XJ, JS, TR, WH, and HW aided with the analysis of the data. XY supervised the experimental design, data analysis, and manuscript writing. SP aided with the analysis of the data and drafting of the manuscript. All authors read and approved the final manuscript.

## Conflict of Interest Statement

The authors declare that the research was conducted in the absence of any commercial or financial relationships that could be construed as a potential conflict of interest.

## Footnotes

1 ftp://ftp.ncbi.nih.gov/genomes/Homo_sapiens

2 ftp://ftp.ncbi.nih.gov/genomes/Homo_sapiens/ARCHIVE/ANNOTATION_RELEASE.105

3 https://4dgenome.research.chop.edu/

4 ftp://ftp.ncbi.nlm.nih.gov/blast/db/

5 https://www.ncbi.nlm.nih.gov/protein

6 http://bioinformatics.psb.ugent.be/webtools/Venn/,Bioinformatics

7 http://www.interactivenn.net/

8 http://www.real-statistics.com/

9 https://www.ncbi.nlm.nih.gov/gene/5747
